# Ductus Venosus Agenesis and Portal System Anomalies—Association and Outcome

**DOI:** 10.3390/biology11040548

**Published:** 2022-04-01

**Authors:** Rodica Daniela Nagy, Nicolae Cernea, Anda Lorena Dijmarescu, Maria-Magdalena Manolea, George-Lucian Zorilă, Roxana Cristina Drăgușin, Sidonia Cătălina Vrabie, Laurențiu Mihai Dîră, Ovidiu Costinel Sîrbu, Marius Bogdan Novac, Nicoleta Alice Marinela Drăgoescu, Mihaela Gheonea, George Alin Stoica, Răzvan Grigoraș Căpitănescu, Dominic-Gabriel Iliescu

**Affiliations:** 1 Doctoral School, University of Medicine and Pharmacy of Craiova, 200349 Craiova, Romania; rodica.nagy25@gmail.com (R.D.N.); laurentiu.dira@yahoo.com (L.M.D.); 2 Department of Obstetrics and Gynecology, University Emergency County Hospital, 200642 Craiova, Romania; nicolae.cernea@gmail.com (N.C.); roxy_dimieru@yahoo.com (R.C.D.); ovidiusoc@yahoo.com (O.C.S.); razvan.capitanescu@gmail.com (R.G.C.); dominic.iliescu@yahoo.com (D.-G.I.); 3 Department of Obstetrics and Gynecology, University of Medicine and Pharmacy of Craiova, 200349 Craiova, Romania; lorenadijmarescu@yahoo.com (A.L.D.); manoleamaria@gmail.com (M.-M.M.); sidocatalina@yahoo.com (S.C.V.); 4 Ginecho Clinic, Medgin SRL, 200333 Craiova, Romania; alin.stoica76@gmail.com; 5 Department of Obstetrics and Gynecology, Clinical Municipal Hospital “Filantropia” of Craiova, 200143 Craiova, Romania; 6 Department of Anesthesia and Intensive Care, University of Medicine and Pharmacy of Craiova, 200349 Craiova, Romania; mariusnovac2005@yahoo.com (M.B.N.); alice.dragoescu@yahoo.com (N.A.M.D.); 7 Department of Anesthesia and Intensive Care, Clinical Municipal Hospital “Filantropia” of Craiova, 200143 Craiova, Romania; 8 Department of Anesthesia and Intensive Care, University Emergency County Hospital, 200642 Craiova, Romania; 9 Department of Neonatology, University Emergency County Hospital, 200642 Craiova, Romania; drmgheonea@yahoo.com; 10 Department of Neonatology, University of Medicine and Pharmacy of Craiova, 200349 Craiova, Romania; 11 Department of Pediatric Orthopedic Surgery, University of Medicine and Pharmacy of Craiova, 200349 Craiova, Romania; 12 Department of Pediatric Orthopedic Surgery, University Emergency County Hospital, 200642 Craiova, Romania

**Keywords:** agenesis of ductus venosus, portal venous system anomalies, outcome

## Abstract

**Simple Summary:**

We have limited information regarding the association and implications of portal venous system (PVS) anomalies in agenesis of ductus venosus (ADV) cases. Few cases of PVS malformations have been reported during fetal life apart from ADV. To the best of our knowledge, this is the first study designed to evaluate the prenatal incidence of ADV and PVS anomalies in the second- and third-trimester anomaly scan experience of a tertiary unit. PVS anomalies were found more frequently than previously reported. We found PVS anomalies in one-third of the ADV cases, while ADV was present in 85.7% of the PVS anomalies. Our data suggest that PVS development is ductus venosus-dependent. PVS anomalies worsened the outcome of ADV cases in our study, and the vast majority of ADV cases that were associated with a normal PVS presented a favorable outcome. The presence of additional fetal anomalies is the best predictor for the outcome of DVA cases. However, an easier evaluation of PVS represents a powerful predictor for ADV cases and addresses the long-term prognosis.

**Abstract:**

To evaluate the prenatal diagnosis of agenesis of ductus venosus (ADV) and portal venous system (PVS) anomalies and describe the outcome of these cases, either isolated or associated. We evaluated the intrahepatic vascular system regarding the presence of normal umbilical drainage and PVS characteristics in the second and third trimester of pregnancy. The associated anomalies and umbilical venous drainage were noted. Follow-up was performed at six months follow-up. Ultrasonography was performed in 3517 cases. A total of 19 cases were prenatally diagnosed: 18 ADV cases, seven abnormal PVS cases, and six associations of the two anomalies. We noted an incidence of 5.1‰ and 1.9‰ for ADV and PVS anomalies, respectively. Out of the 18 ADV cases, 27.7% were isolated. Five cases (26.3%) presented genetic anomalies. PVS anomalies were found in 33.3% of the ADV cases. ADV was present in 85.7% of the PVS anomalies. DV and PVS abnormalities were found with a higher than reported frequency. Normal DV is involved in the normal development of the PVS. Additional fetal anomalies are the best predictor for the outcome of ADV cases. Evaluation of PVS represents a powerful predictor for ADV cases and addresses the long-term prognosis.

## 1. Introduction

The ductus venosus (DV) is supposed to play an essential role in fetal circulation because it allows the oxygenated placental blood to bypass the liver and enables preferential flow via the right atrium and foramen ovale to the left atrium. Furthermore, normal umbilical flow the DV is involved in the normal development of the intrahepatic portal venous system (IHPVS) [1]. 

The lack of ‘critical anastomoses’ between the portal and umbilical venous system and hepatic–systemic venous system results in agenesis of ductus venosus (ADV) and shunting of umbilical blood through an aberrant vessel. Between 1 in 2532 to 1 in 556 fetuses had various incidences of ADV [2,3]. On the other hand, anomalies of the portal venous system (PVS) have been rarely reported prenatally, as per anecdotal reports: almost 2.5 postnatal cases per year over the last 30 years have been reported [4]. Furthermore, more than 22% of patients had associated congenital heart disease [4].

Two main types of ADV have been described, depending on the course and connection of the umbilical vein (UV): intra- and extrahepatic [5]. 

Different outcomes of ADV cases have been reported, related either to their association with other fetal abnormalities and the nuchal translucency (NT) measurement, the development of the PVS, the type of umbilical shunt, or the caliber of the shunt [6,7,8,9]. However, the isolated nature of the anomaly is best associated with a favorable outcome [3].

Total portal venous system agenesis (TPVSA) occurs due to the vitelline veins (VV) failure to transform into the portal system, leading to a failure to form the critical anastomosis with the hepatic sinusoids or UVs [10]. Isolated TPVSA is rare and frequently reported in association with other fetal anomalies such as heterotaxy, polysplenia, congenital heart defects, Goldenhar syndrome, and chromosomal anomalies.

Partial portal venous system agenesis (PPVSA) occurs because of partial failure to form critical anastomoses and represents a benign condition with a better outcome, as it rarely associates with other malformations. Furthermore, the intrahepatic shunt ratio may influence the circulatory system, with intrauterine growth restriction [11].

The failure to develop critical anastomosis between the UV and vitelline venous system represents the link between the association of ADV and total or partial agenesis of the portal venous system. The absence of DV determines abnormal hemodynamics as there is a “steal” effect with a decreased intrahepatic flow, which may lead to the failure of the vitelline veins to transform into the portal system [8]. Postnatal there are important implications of PVS anomalies with potential later complications, in adulthood. ADV and PVS anomalies have a common origin, and the prognosis is variable, given the physiological importance of the two venous systems. 

We have limited information regarding the implications of TPVSA or PPVSA association in ADV cases. However, few cases of PVS anomalies have been reported during fetal life apart from ADV cases. 

The guidelines do not recommend the routine investigation of PVS features and ductus venosus confirmation [12,13]. Anomalies of these systems are most likely underdiagnosed prenatally. Therefore, this study evaluated the incidence, association, and outcome implications of portal system anomalies and ADV.

## 2. Materials and Methods

This prospective cohort study was designed and conducted in the regional tertiary center of Craiova (Prenatal Unit of University Emergency County Hospital Craiova and GINECHO Clinic), with approximately 3500 births per year, between September 2018 and May 2021. The Ethics and Scientific Deontology Commission of the University of Medicine and Pharmacy, Craiova, Romania, issued the ethical approval of this research project in agreement with the ethical principles of the Helsinki declaration and University Code of Ethics on the proper conduct of research. All women provided written informed consent before the ultrasound examination.

**Figure 1:**
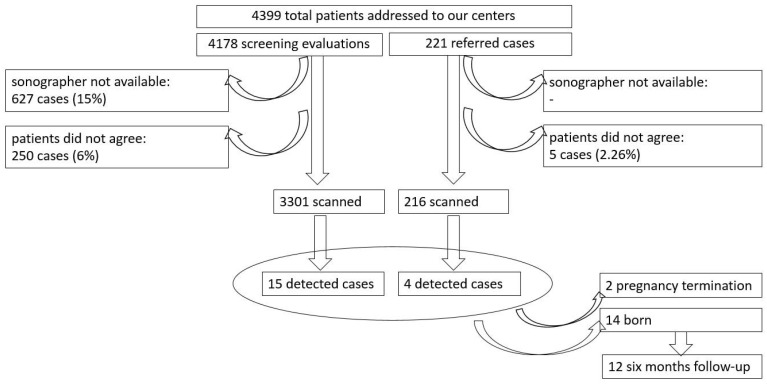
Flow chart summarizing the study group.

A systematic color Doppler assessment of the intrahepatic venous system, including DV and portal system, was proposed to all pregnant women in the second or third trimester. The fetal venous evaluation scan was performed simultaneously with the scheduled ultrasound. The patients were included in the study consecutively, depending on their acceptance and the sonographers’ availability. Overall, trained physicians were available for almost 85% of the scans (Figure 1).

The cases referred for fetal anomalies and multiple pregnancies were not excluded from the study. Re-evaluation was offered to all cases where an evaluation could not be completed because of the patient body habitus or fetal position. We excluded from the study the cases that did not comply with the protocol and the patients lost for follow-up before the ultrasound scan was completed. 

**Figure 2:**
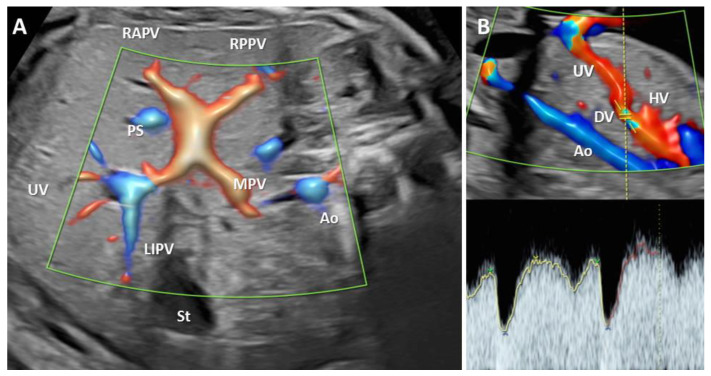
The typical aspect of the portal venous system (PVS) and ductus venosus (DV). (**A**): Transverse plane of the fetal abdomen, with high-definition directional power Doppler, applied to demonstrate the normal L-shaped UV confluence and PVS features. (**B**): High-definition power flow Doppler image of the fetal circulation showing typical Doppler waveforms in DV. UV, umbilical vein; RAPV, anterior branch of the right portal vein; RPPV, the posterior branch of the right portal vein; LIPV, left portal vein inferior branch; St stomach; Ao aorta; HV hepatic vein.

Ultrasound (US) examinations were performed trans-abdominally using Voluson E10 and E8 (General Electric System, GE Healthcare, Zipf, Austria) and Philips Elite Epiq (Bothell, USA) ultrasound machines, equipped with a 4–8-MHz curvilinear transducer. Only certified sonographers with a minimum of three years of experience in obstetrical ultrasound were involved in the study. The scan protocol followed the international guidelines 12. Additionally, DV was ascertained in the longitudinal view of the upper fetal abdomen, with the image magnified so that the fetal thorax and abdomen occupied the whole screen. Also, we evaluated the PVS features in axial abdominal planes, magnified to occupy half of the screen. We identified the L-shaped portal sinus (PS), the junction of PS with the right and left portal veins, and the main portal vein, which runs from the left side between the stomach and the descending aorta (Figure 2) [14]. Color Doppler assessment was performed following the ALARA principle, with the mechanical and thermal indices kept as low as possible. The abnormal scan findings underwent a second opinion: two experienced maternal-fetal specialists performed a supplementary evaluation in ADV, PPVSA, or TPVSA cases. The associated anomalies and umbilical venous drainage were noted in all cases.

Following confirmation, an interdisciplinary team counseled the women regarding the long-term prognosis of the case and proposed appropriate management. The counseling team included geneticists, neonatal pediatricians, a pediatric cardiologist, a pediatric surgeon, and maternal–fetal specialists. Genetic counseling was proposed to all ADV cases, and genetic testing was performed in all cases, including fluorescence in-situ hybridization (FISH) and karyotype (standard). Array-comparative genomic hybridization (array-CGH) was reserved for selected cases at the geneticists’ indication. Maternal characteristics and medical history were recorded. In all live births with ADV or PVS anomalies, a detailed postnatal evaluation was performed and also, a follow-up evaluation at six months was proposed. In addition, a perinatal autopsy was offered in stillbirths and the cases that underwent pregnancy termination.

We searched international databases (PubMed, Scopus, and Ebsco) for ADV studies where information regarding the PVS was provided. Also, we searched for research regarding the prenatal detection of abnormal PVS cases, where the umbilical drainage was communicated. The following keywords were used: agenesis/absence of ductus venosus, prenatal/fetal portal system, total/partial portal agenesis. 

Our main objective was to evaluate the association and outcome implications in portal system anomalies and ADV cases. The secondary objectives were to calculate the ADV and PVS anomalies incidence, identify the prognostic role of PVS development in ADV cases, determine the anomalies of umbilical drainage in TPVSA and PPVSA cases, and compare our results with published literature.

Statistical analyses were carried out using IBM SPSS 19.0 (BM SPSS Inc., New York, NY, USA). The chi-square test or the Fisher Exact test (when minimum expected count was less than 5) was used to compare proportions between groups. *p*-values less than 0.05 were considered statistically significant. There was no need for statistical methods to prevent confusion.

## 3. Results

**Figure 3:**
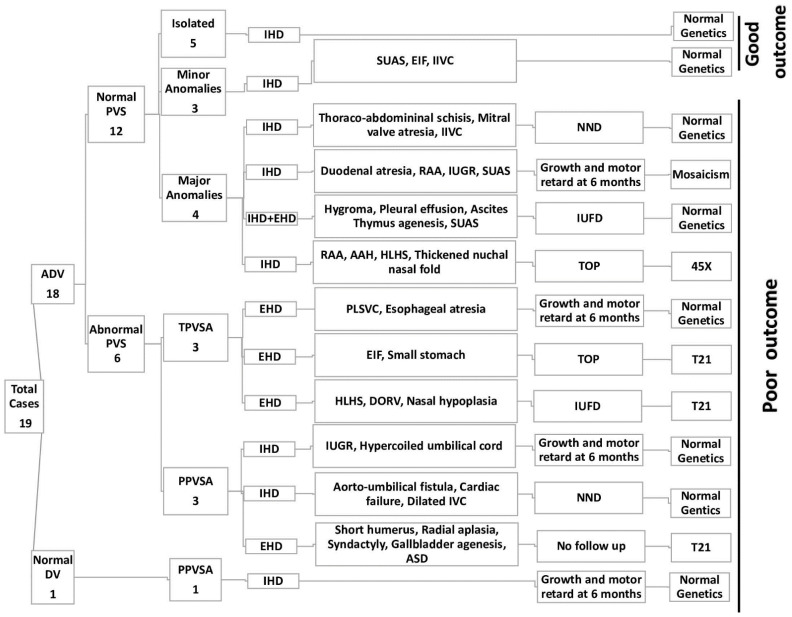
Schematic representation of the 19 cases and the associated anomalies with sonographic findings and outcome. ADV: agenesis of ductus venosus; PVS: portal venous system; IHD: intrahepatic drainage; EHD: extrahepatic drainage; SUAS: single umbilical artery; EIF: echogenic intracardiac focus; IIVC: interrupted inferior vena cava; NND: neonatal death; RAA: right aortic arch; IUGR: intrauterine growth restriction; IUFD: intrauterine fetal death; AAH: aortic arch hypoplasia; TOP: termination of pregnancy; TPVSA: total portal venous system agenesis; PPVSA: partial portal venous system agenesis; PLSVC: persistent left superior vena cava; DORV: double outlet of the right ventricle; IVC: inferior vena cava; ASD: atrial septal defect; DV: ductus venosus; HLHS: Hypoplastic left heart syndrome; T21: trisomy 21; 45X: Turner syndrome.

During the study period, the second and third trimester assessments of DV and PS were carried out in 3517 pregnancies. A total of 216 cases were referred to our tertiary center for suspected anomalies, 6.14% of the studied population. A total of 19 cases with ADV and PVS anomalies were diagnosed: 12 cases with ADV and normal PVS, three cases with ADV and TPVSA, three cases with ADV and PPVSA, and one case of PPVSA with typical DV was identified, giving an incidence of 5.1‰ for ADV (18 cases) and 1.9‰ for PVS anomalies (seven cases) (Figure 3). Four of the 19 cases (21%) were referred from other centers for a second opinion or final diagnosis in cases suspected of fetal anomalies. The median maternal age was 29 years for the DV and PVS anomalies group. The medical history of the pregnant women was irrelevant (one premature birth and one preeclampsia). 

The prenatal findings and outcomes of the 19 cases are summarized in Table 1 and Figure 3.

### 3.1. ADV with Normal PVS

**Figure 4:**
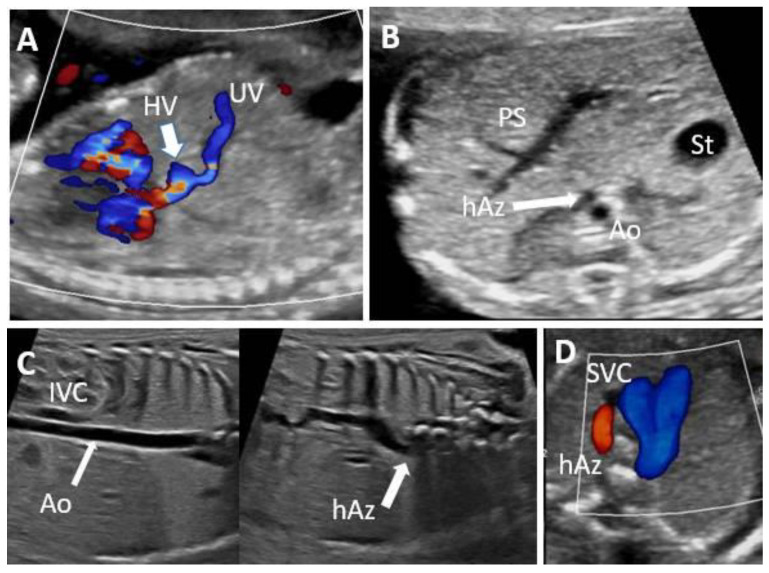
Agenesis of ductus venosus (ADV) in a case with interrupted inferior vena cava and narrow intrahepatic shunt and typical portal venous system at 20 weeks of gestation (case 3). Vascular abnormalities are detected in the upper abdomen. (**A**): Color Doppler imaging, showing ADV with umbilical vein drainage into a hepatic vein. (**B**): Transverse abdominal plane at the level of the portal confluent, with normal appearance. (**C**): Coronal view of the abdomen and thorax showing absence of the hepatic segment with hemiazygos continuation. (**D**): Duplex evaluation (greyscale and color Doppler) shows hemiazygos vein drainage into the superior vena cava evident in the three-vessel and trachea view. UV umbilical vein, PS portal system, IVC inferior vena cava, HV hepatic vein, SVC superior vena cava, Ao aorta; hAz hemiazygos, St stomach.

**Figure 5:**
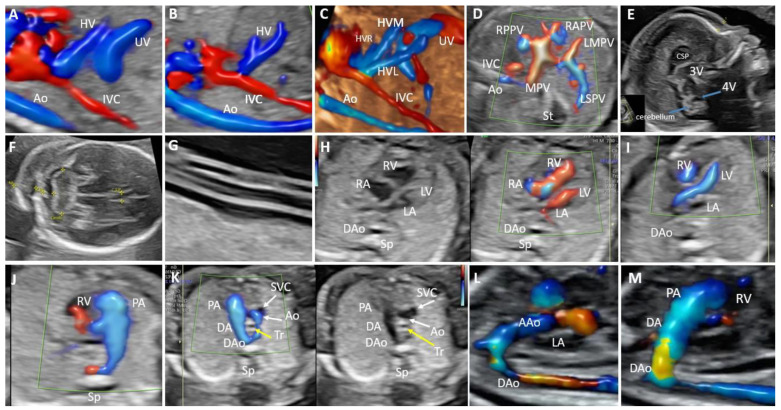
Agenesis of the ductus venosus in a case with a typical portal venous system and down-displacement of the hepatic efferent system (case 19). (**A**): Color Doppler imaging shows the umbilical vein draining into the portal system in the sagittal plane without giving rise to the ductus venosus. (**B**): Sagittal plane and Color Doppler demonstration of down-displacement of the hepatic efferent system. (**C**): 4D STIC reconstruction, showing in the sagittal plane the abnormal drainage of the umbilical vein and the lower insertion of the hepatic veins below the prediaphragmatic infundibulum. (**D**): The abdomen’s transverse view shows the typical portal venous system. (**E**): Midsagittal view fetal head on two-dimensional ultrasound imaging shows the increased prenasal thickness and normal brain features. (**F**): Transcerebellar plane of the fetal head showing thicken nuchal fold. (**G**): Two-dimensional ultrasound imaging showing hypocoiled umbilical cord. (**H**): 2D and Color Doppler image of the four-chamber view showing disproportion between the right and left ventricles. (**I**): Left ventricular outflow tract view showing a small aorta. (**J**)**:** Right ventricular outflow tract view showing a normal pulmonary artery bifurcation. (**K**): Sonographic image in 3-vessel trachea view showing the position of the aortic arch in comparison to the trachea (yellow arrow); the trachea is located between the right-sided arch and left-sided ductus forming a U-shaped loop. (**L**): Sagittal view of the hypoplastic right aortic arch. (**M**): Ductal arch view. The ductus arteriosus connects the main pulmonary artery to the descending aorta, forming a hockey stick-shaped arch. HV: hepatic vein; Ao: aorta; IVC: inferior vena cava; UV: umbilical vein; HVL: left branch of the hepatic vein; HVM: medial branch of the hepatic vein; HVR: right branch of the hepatic vein; RAPV: anterior branch of the right portal vein; RPPV: posterior branch of the right portal vein; LMPV: medial branch of the left portal vein; LSPV: superior branch of the left portal vein; MPV: main portal vein; St: stomach; 3V: third ventricle; 4V: fourth ventricle; CSP: cavum septum pellucidum; NF: nuchal fold; RV: right ventricle; RA: right atrium; LA: left atrium; LV: left ventricle; DAo: descending aorta; Sp: spine; PA: pulmonary artery; DA: ductus arteriosus; Tr: trachea; SVC: superior vena cava; AAo: ascending aorta.

**Figure 6:**
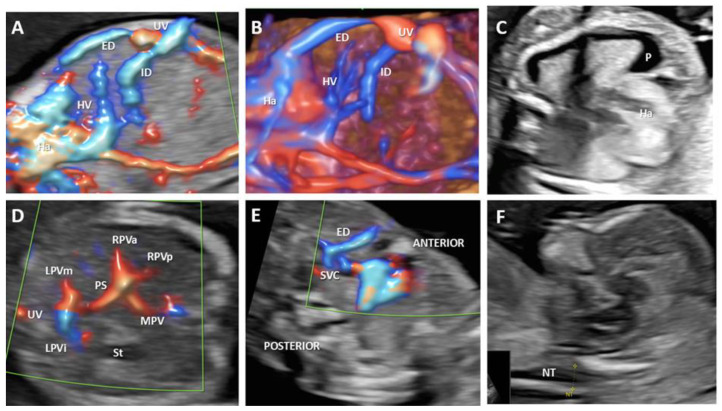
Agenesis of the ductus venosus with complex drainage of the umbilical vein: intrahepatic umbilico-portal drainage, and extrahepatic drainage, into the superior vena cava at 14 weeks of gestation (case 4). (**A**): Longitudinal plane of the fetal abdomen, color Doppler evaluation, showing the absence of ductus venosus and umbilical drainage. (**B**): 3D evaluation showing the abnormal umbilical vein drainage: umbilico-portal and into superior vena cava. (**C**): Transverse view at the thorax level showing the presence of pleural effusion. (**D**)**:** Transverse view at the abdomen level showing a normal portal venous system (**E**): Three-vessel view showing the extrahepatic drainage of the umbilical vein into the superior vena cava. (**F**): Median section used for the sonographic measurement of nuchal translucency thickness. UV—umbilical vein. SVC—superior vena cava. ED—extrahepatic drainage. ID—intrahepatic drainage. HV—hepatic vein, Ha—the heart. P—pleural effusion. MPV—main portal vein. PS—portal sinus. RPVa—the anterior branch of the right portal vein. RPVp—the posterior branch of the right portal vein. LPVi—left portal vein inferior branch; LPVm—left portal vein medial branch. St—stomach. NT—nuchal translucency.

Two-thirds (12 cases, 66.6%) of the ADV cases presented a normal PVS. Of these 12 cases, only five (41.6%) were isolated (cases 5, 6, 7, 8, 9). Three cases (25%) were associated with minor anomalies (single umbilical artery, echogenic intracardiac foci or isolated interrupted inferior vena cava (cases 1, 2, 3; case 3 is presented in Figure 4) and four cases (33.3%) associated with major malformations as thoracoabdominal schisis, mitral valve atresia, interrupted inferior vena cava (case 16), duodenal atresia, right aortic arch, IUGR, single umbilical artery (case 18), fetal cardiac asymmetry, aortic arch hypoplasia, thickened nuchal fold (case 19, Figure 5), hygroma, ascites, pleural effusion (case 4, Figure 6). An adverse outcome was noted in all cases associated with major malformations: one pregnancy termination (TOP), one intrauterine fetal death (IUFD), one neonatal death, and one growth and motor retardation at six months follow up.

### 3.2. ADV with PVS Partial or Total Agenesis

**Figure 7:**
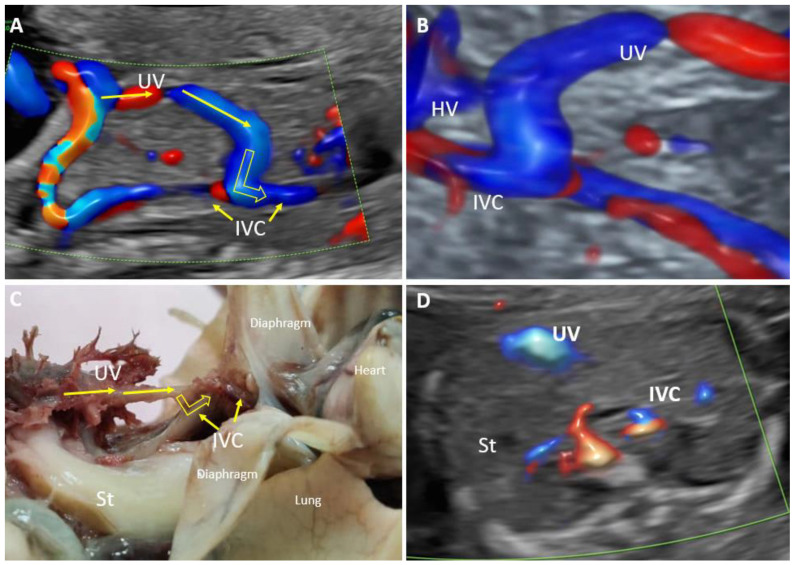
Agenesis of the ductus venosus with a wide extrahepatic shunt to the inferior vena cava at 20 weeks of gestation (case 11). (**A**): Longitudinal plane of the fetal abdomen, color Doppler evaluation, showing the absence of ductus venosus and a wide umbilical shunt directed to the inferior vena cava. (**B**): 3D evaluation shows abnormal umbilical vein drainage into the inferior vena cava. (**C**): Pathological examination showing the drainage of the umbilical vein into the inferior vena cava. (**D**): Transverse view at the level of insertion of the umbilical cord showing the absence of the portal venous system and persistent small stomach (white arrow) UV—umbilical vein. IVC—inferior vena cava. HV—hepatic vein.

**Figure 8:**
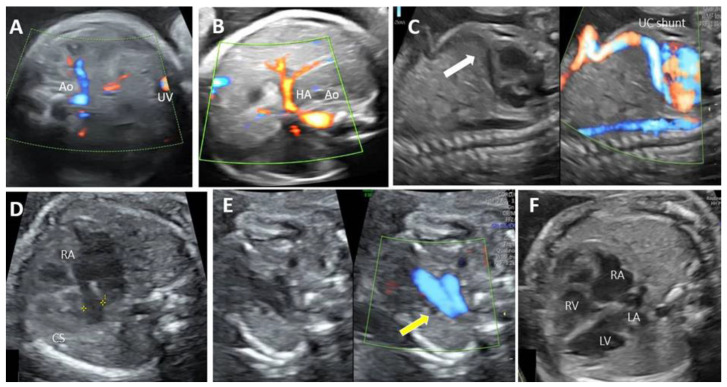
Agenesis of ductus venosus (ADV) with wide extrahepatic cardiac shunt and absence of portal system at 26 weeks of gestation (case 10). (**A**): Transverse view at the level of insertion of the umbilical cord showing absence of the intrahepatic segment of the UV and portal venous system. (**B**): Transverse section of the upper abdomen on the greyscale and color Doppler assessment, with the umbilical cord insertion, the prominent hepatic artery, and no afferent liver venous perfusion. (**C**): Longitudinal plane of the fetal abdomen and thorax, showing the extrahepatic course of the UV and the wide drainage toward the base of the heart, into the right atrium. (**D**): Four chamber view with dilated coronary sinus. (**E**): Three vessel planes in Duplex evaluation (greyscale and color Doppler) show four vessels: the ductal and aortic arterial arches and their confluence at the left of the spine, right and persistent left superior vena cava (yellow arrow). (**F**): Four chamber view with enlarged right atrium due to PLSVC. UV—umbilical vein. UC—umbilico-cardiac. RA—right atrium. LA—left atrium. RV—right ventricle. LV—left ventricle. PLSCV—persistent left superior vena cava. CS—coronary sinus HA—hepatic artery.

Three of the ADV cases (16.6%) were accompanied by TPVSA. In case 11, the couple requested TOP as genetic evaluation revealed Trisomy 21 (Figure 7, Video 1). IUFD occurred in case 17, associated with hypoplastic left heart syndrome and double outlet of the right ventricle. In one case, we confirmed persistent left superior vena cava and esophageal atresia at birth, which was successfully repaired (case 10, Figure 8, Video 2). However, in this case, the outcome is impaired by retarded growth and motor deficit at six months after birth. In all TPVSA cases, we noted extrahepatic umbilical drainage.

From the 18 ADV cases, three cases (16.6%) were associated with PPVSA. Two of them presented intrahepatic drainage of the UV (cases 12 and 14). In case 12, we noted severe symmetrical growth restriction, hypercoiled umbilical cord, normal uterine and umbilical flow velocities, and normal genetics. The outcome was good at birth, but severe deficits were noted at six months.

### 3.3. Umbilical Drainage in PVS Agenesis Cases

We investigated the pattern of the umbilical drainage in ADV cases associated with abnormal PVS. Extrahepatic shunting was noted in all three TPVSA cases and one PPVSA case (4/6; 66.6%). The other two PPVSA cases had intrahepatic drainage. We also noted intrahepatic shunting in all 12 ADV cases with normal PVS, except one case with a complex intra- and extrahepatic drainage (case 4).

**Figure 9:**
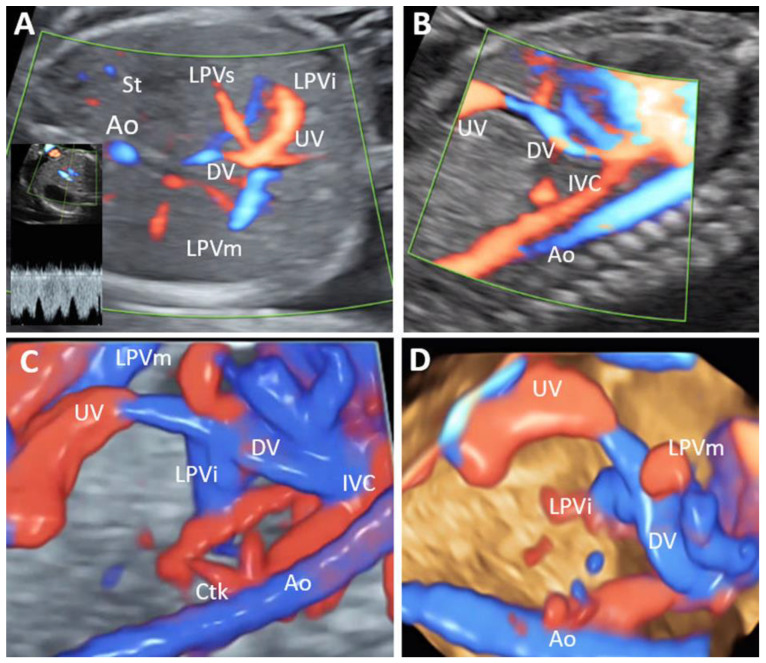
Partial portal venous system agenesis (PPVSA) (Case 13–20 weeks of gestation). (**A**): Transverse view of the fetal abdomen, showing the confluence of the umbilical vein (UV) with left portal vein (LPV) branches, but the absence of a normal portal sinus and right portal vein. (**B**): Sagittal view showing the presence of ductus venosus. (**C**,**D**): Sagittal view showing in 3D the presence of ductus venosus and left portal vein branches. Ao—aorta; IVC, inferior vena cava; LPVi—left portal vein inferior branch; LPVm—left portal vein medial branch; LPVs—left portal vein superior branch; UV—umbilical vein; DV—ductus venosus; Ctk—celiac trunk; St—stomach.

PPVSA associated ADV in 75% of the cases. In one case only, the abnormal PVS (PPVSA) associated a normal umbilical drainage and ductus venosus (Figure 9).

### 3.4. Umbilical Drainage and Pregnancy Outcome

The characteristics of the umbilical drainage were investigated in all ADV cases concerning the fetal outcome.

In 14 cases (73.6%), intrahepatic shunting was described. We noted a good outcome at birth and six-months follow-up in the group of isolated cases or associated with minor anomalies (eight cases). Among the cases with intrahepatic drainage and major anomalies, two (14.2%) associated intrauterine growth restriction (IUGR). In the group of intrahepatic shunting, we noted two NNDs (14.2%), one TOP (7.1%), and three survivors who presented growth and motor retardation at six months follow up (21.4%).

An extrahepatic shunt was diagnosed in four cases (21%), and a multiple, complex shunting of the umbilical flow (portal and extrahepatic—superior vena cava) was found in one case. All these cases associated major anomalies and a poor outcome: two IUFDs, one TOP, and one case with growth and motor retardation at six months follow-up. Unfortunately, the follow-up was not performed in one case as the patient did not further refer to our center.

### 3.5. Genetics

A genetic evaluation was performed in all cases, and chromosomal anomalies were identified in five cases (27.7%): most fetuses were affected by trisomy 21 (three cases). In addition, one mosaicism case and one Turner Syndrome were also detected.

### 3.6. What Is the Best Predictor for ADV Cases Outcome?

The measure of association, or correlation, between two categorical attributes, such in our case, can be computed using Pearson’s Chi-Square test [15]. The null hypothesis is H_0: the variables are not correlated with each other. A value larger than 0.05 for the corresponding *p*-level implies the acceptance of the null hypothesis. The results of the test are presented in Table 2.

We can see from Table 2 that the only variables that are correlated with the DVA outcome are the additional fetal anomalies, with a corresponding *p*-level of 0.0075, and PVS, with a lower yet significant level, 0.027.

## 4. Discussion

The second and third trimester ADV rate is almost three-fold higher than PVS anomalies. The incidence of ADV cases in our study was 5.1‰, higher than that reported previously. The incidence of PVS anomalies was 1.9‰, also more frequent than in postnatal studies, where only 83 cases were reported in a relatively recent systematic review [4]. Probably, this is due to the intrauterine deaths of the affected fetuses and the fact that the evaluation of the portal system is not part of the standard protocol during perinatal autopsy.

To the best of our knowledge, this is the first study with a protocol designed to evaluate the prenatal incidence of these anomalies in all pregnant populations scanned in the second and third trimesters. Still, these prenatal incidence figures should not be generalized for the entire gestation, for they may be biased because of the fetal demise or pregnancy terminations in some first trimester cases. Therefore, we did not search for the incidence in the first trimester, which may be higher. We noted a much lower incidence at birth, of 3.6‰ and 0.8‰ for ADV and PVS anomalies, respectively. Also, we describe a partial referral population examined in a tertiary center. Therefore, we cannot state that the incidence figures represent the actual incidence of the anomaly. Our study population’s vast majority consisted of standard screening anomaly scan evaluations. However, we consider our group a medium-risk population because we frequently examine cases referred for cardiovascular abnormalities in our tertiary center, which are known to have a higher incidence of ADV [2,3]. A total of 6% of the studied patients were referred to our unit, and in this group, we detected almost one-quarter of the abnormal DV or PVS cases (21%). Thus, sonographers who work in such tertiary reference centers should be aware that they may face these vascular anomalies more frequently than reported in the literature.

About one quarter (27.7%) of the ADV cases were isolated in our series, much less than reported in the literature (35–59%) [16]. In half of the cases associated with major structural defects, cardiovascular abnormalities were present. A high percentage is also reported in the literature, 42% [7]. The most frequently observed anomalies were hydrops, pleural effusion, and cardiac failure due to poor fetal hemodynamics.

A general conclusion of our study and literature is that the cases that showed adverse neonatal outcomes were not isolated, and major anomalies were present. Therefore, the true importance of PVS prenatal investigation would be for proper postnatal monitoring to detect the medium- and long-term implications of portal system development. The results will offer important information for the prenatal investigations and counseling in ADV cases.

Chromosomal abnormalities have been reported frequently associated with ADV, between 17% and 24% [17,18]. In our group of ADV cases with genetic evaluation, 27.7% had an abnormal genetic result. Perhaps a more extensive genetic assessment should be recommended, given the rate of associated malformations and lack of literature on the ADV genetic associations.

PVS anomalies were present in one-third of the ADV cases in our study and as many as 50% of cases in the reviewed literature [16]. In ADV cases associated with PVS, the portal system agenesis was total in half of our cases and 71% in a previous series of nine cases with PVS anomalies [10]. Based on the published studies, we believe that abnormalities in the portal venous system are more likely to occur along with abnormalities of the ductus venosus due to the connections established during embryology. Normal umbilical flow via DV seems to be involved in the normal development of the intrahepatic portal venous system (IHPVS) [1,5]. The hypothesis that PVS development is DV-dependent appears valid, as ADV was associated with 85.7% of the PVS anomalies from our reported cases and 77.7% in the previous series [10]. Only PPVSA was encountered when umbilical drainage was normal in our study and the literature [16].

In one-third of the cases, ADV fetuses with normal PVS were associated with major anomalies, and all ended with a poor outcome. The ADV cases associated with PVS anomalies had a higher rate of major morphological and genetic anomalies (83.3%), with a generally poor outcome. Therefore, the sonographers should search for associated anomalies when PVS appears abnormal. PVS anomalies (total or partial) worsened the outcome of ADV cases in our study, as no case presented a final good outcome.

Conversely, the vast majority of ADV cases with normal PVS presented a favorable outcome in our group (66.6%) and in the literature as well (80–100%) [16]. Still, we should not generalize this conclusion, as major structural malformations may be found in cases with normal PVS (case 4, Figure 5), which significantly influence the neonatal outcome.

Umbilical drainage represents a critical prognostic feature described in the literature [6]. In our study and the previous reports, intrahepatic drainage is more common than extrahepatic ones. Almost three-quarters of the affected fetuses (73.6%) had intrahepatic drainage of the umbilical vein. Previous studies suggest that extrahepatic drainage involves a poor outcome, contrary to intrahepatic [10]. We are partially in line with the literature as all extrahepatic shunts from our series had an adverse outcome. However, our data show that the intrahepatic shunts indeed carry a better prognosis only if isolated or associated with minor abnormalities.

Significant postnatal implications of portal system anomalies have been reported. The absence of hepatotrophic factors supplied by portal venous blood may determine liver atrophy. In addition, a metabolic disorder, such as high blood galactose, manifests in the neonatal period, both of which are commonly mild and nonspecific [19]. These conditions explain the neuro-motor deficits displayed in the TPVSA case with an apparently normal outcome at birth. On the other hand, in adulthood, long-term exposure to portal venous deprivation due to PVSA, acute liver failure, or liver nodes were reported [20]. The bypass of unprocessed portal metabolites into the systemic venous system leads to portosystemic encephalopathy (PSE), which shows symptoms more in adults than in children [20,21]. Portopulmonary hypertension (PPH) and hepatopulmonary syndrome (HPS) may also be present in children with agenesis of the portal venous system [22]. Symptomatic cases should almost always be treated, as the symptoms are life-threatening (cardiopulmonary symptoms, liver nodules) or impair the quality of life (encephalopathy). The treatment options include transplantation if there is severe liver failure or on the contrary, in mild liver dysfunction, only a low-protein diet may be indicated. For TPVSA there is no curative treatment beside liver transplantation because the reconstruction of the PVS is impossible [19]. For both ADV and PVS anomalies, some reports strongly suggest that shunts should be closed before a significant complication occurs, such as acute liver failure after dehydration, nonreversible pulmonary hypertension, liver malignancy, or the long-term effects of chronic hyperammonemia on the developing brain [8].

In isolated ADV cases, the postnatal outcome is influenced by the agenesis of the portal venous system as the association may affect the long-term outcome due to severe postnatal complications [5,23,24,25]. Achiron reported four cases of PPVSA and five cases of TPVSA. Only one case of PPVSA was associated with ADV, and the prognosis was good in all four cases. In contrast, all TPVSA cases resulted in a poor outcome, as they were associated with ADV and other structural anomalies [10]. ADV seems to be a problem that affects only the fetal period, while the anomaly or absence of the portal venous system acts as a prognostic factor, with long-term consequences [24]. The poor prognosis of extrahepatic shunts is well-known because of the reported association with hydrops, malformations, and chromosomal syndromes such as trisomy 21 [5]. However, the ADV cases with extrahepatic drainage were associated in our study with maldevelopment of the PVS; therefore, these conditions generally overlap.

Whenever we diagnose ADV, we should consider the possibility of complex umbilical connections. Multiple intra- and extrahepatic shunts are presented in our series (case 4) and previous reports [26]. However, these are the only two reported cases; therefore, the pattern of this condition should be further investigated.

The true importance of detecting ADV during the fetal anomaly scan is questionable since all adverse outcomes were registered in non-isolated cases, where additional anomalies were present. Therefore, the functional importance of DV may be overestimated in the classic literature and textbooks, since in our series and literature as well, isolated ADV associates with a normal newborn outcome. However, portal system normality may represent an important medium- and long-term outcome predictor in ADV cases. Cases 10 and 12 of our series provide good arguments for this statement, as during early infancy, the apparently good neonatal outcome changed significantly when severe growth restriction and neuro-motor deficiencies became apparent. Our observation, similar to those described in other reports [10], confirms that an apparently normal outcome at birth in cases with partial or total agenesis of the PVS should be re-evaluated due to the potential later complications. Counseling in ADV cases should consider the PVS development because of the role in potential long-term complications that may occur when the portal system is affected [27,28].

The presence of additional fetal anomalies represents the most powerful predictor for DVA outcome, but also PVS normality reaches statistical significance. We may add that PVS evaluation is much easy than a fetal anomaly scan. Our previous reports demonstrate that even in first trimester, the L-shaped UV confluence can be achieved, that enables the detection of major PVS abnormalities [29]. Therefore, this parameter may be more suitable in some settings as a predictor for ADV cases. And not to forget, PVS evaluation also considers the long-term prognosis.

The limited postnatal follow-up represents a limitation of ADV’s previous reports and the present studies. A long-term follow-up is needed because the portal system-related conditions may not be evident in the newborn or at the six-month evaluation, but later in the adult life [24]. We should further monitor the affected children because this is the only way to demonstrate the importance of the ductus venosus and the implications of the isolated ADV. As stated before, there is a conflict between experimental studies that state the functional importance of DV and clinical studies, showing a normal outcome in isolated ADV cases [30].

The lack of detailed genetic analysis represents another limitation of our study and the literature. Therefore, molecular array-CGH evaluation should be completed by trio-exome, because some ADV cases may associate with Noonan Syndrome or other monogenic diseases not detectable on the array-CGH investigation. Further, some postnatal delays can be attributed to mutations or syndromic causes.

Our work’s strength and originality consist of the concomitant standard evaluation of umbilical connections and portal system normalcy in the general population, the correlations of the two conditions, and the postnatal follow-up to detect some of the potential implications of DV and PVS anomalies.

## 5. Conclusions

DV and PVS abnormalities were found with a higher than reported frequency in the scanned population. PVS anomalies were found frequently in ADV cases, while ductus venosus was absent in all TPVSA cases. We believe that ADV diagnosis should automatically lead to the PVS evaluation because portal anomalies represent a significant outcome predictor of ADV cases. TPVSA is strongly correlated with extrahepatic umbilical shunting and associates with a poor outcome regardless of genetic and morphological associations.

Furthermore, the absence or underdevelopment of the portal system worsens the outcome, and parents should be counseled on the possibility of long-term complications for a proper decision on the continuation of the pregnancy. Longitudinal studies are needed after birth, as a normal neonatal outcome is insufficient.

When the absence of ductus venosus is isolated, and the portal system is developed, a good outcome should be expected. However, a careful evaluation is required to detect signs of fetal maldevelopment and hydrops in all cases.

Our data confirm that PVS development is ductus venosus-dependent. Furthermore, PVS anomalies worsen the outcome of ADV cases, while the vast majority of ADV cases associated with a normal PVS presented a favorable outcome.

The presence of additional fetal anomalies is the best predictor for the outcome of DVA cases. However, an easier evaluation of PVS represents a powerful predictor for ADV cases and addresses the long-term prognosis.

Our study provides additional clinical insight into prenatal ultrasound predictors of ADV outcome, which may help prenatal counseling and postpartum monitoring.

## Figures and Tables

**Table 1:** Ultrasound findings and outcome in the cases with absent ductus venosus or abnormal portal system development.

No	MA	GA	DV	Umbilical Drainage	PVS	Additional Sonographic Findings	Genetics	Outcome
1	27	18	ADV	intrahepatic	N	SUA	Normal karyotype	Good at birth and at 6 months follow-up
2	32	18	ADV	intrahepatic	N	EIF	Normal karyotype	Good at birth and at 6 months follow-up
3	30	20	ADV	intrahepatic	N	IIVC	Normal karyotype	Good at birth and at 6 months follow-up
4	28	18	ADV	intrahepatic and extrahepatic	N	Hygroma SUA Thymus agenesis Ascites Pleural effusion	Normal QF-PCR and array CGH	IUFD
5	29	18	ADV	intrahepatic	N	-	Normal karyotype and QF-PCR	Good at birth and at 6 months follow-up
6	25	20	ADV	intrahepatic	N	-	Normal karyotype	Good at birth and at 6 months follow-up
7	38	19	ADV	intrahepatic	N	-	Normal karyotype	Good at birth and at 6 months follow-up
8	28	18	ADV	intrahepatic	N	-	Normal karyotype	Good at birth and at 6 months follow-up
9	36	23	ADV	intrahepatic	N	-	Normal karyotype	Good at birth and at 6 months follow-up
10	27	26	ADV	extrahepatic	TPVSA	PLSVC Esophageal atresia	Normal karyotype	Good at birth Growth and motor retard at 6 months follow-up
11	33	20	ADV	extrahepatic	TPVSA	EIF Small stomach	T21	TOP
12	29	30	ADV	intrahepatic	PPVSA	IUGR Hypercoiled umbilical cord	Normal karyotype	Good at birth Growth and motor retard at 6 months follow-up
13	25	20	Normal	Intrahepatic	PPVSA	-	Normal karyotype	Good at birth Growth and motor retard at 6 months follow-up
14	31	28	ADV	intrahepatic	PPVSA	Aorto-ombilico-hepatic fistula Cardiac failure Dilated IVC	Normal QF-PCR	NND
15	25	24	ADV	extrahepatic	PPVSA	Bilateral short humerus Unilateral absence of the radius Syndactyly Gallbladder agenesis ASD	T21	No follow up
16	34	33	ADV	intrahepatic	N	Thoracoabdominal schisis Mitral valve atresia IIVC	Normal karyotype	NND
17	25	16	ADV	extrahepatic	TPVSA	Hypoplastic left heart syndrome DORV; Nasal hypoplasia	T21	IUFD
18	28	24	ADV	intrahepatic	N	Duodenal atresia SUA Right aortic arch IUGR	mos 47, XY, +mar [15]/46,XY [38].	Growth and motor retard at 6 months follow up
19	26	20	ADV	intrahepatic	N	RAA Fetal cardiac asymmetry Hypocoiled Umbilical Cord AAH Hyperechogenic bowel Thicken Nuchal Fold Increased prenasal thickness	45X	TOP

MA, maternal age; GA, gestational age; DV, ductus venosus; ADV, ductus venosus agenesis; PVS, portal venous system; IVC, inferior vena cava; TPVSA, total portal venous system agenesis; PPVSA, partial portal venous system agenesis; SUA, single umbilical artery; EIF, echogenic intracardiac focus; IIVC, interrupted inferior vena cava; PLSVC, persistent left superior vena cava; TOP, termination of pregnancy; GB, good at birth; IUFD, intrauterine fetal death; IUGR, intrauterine growth restriction; DORV, double outlet of the right ventricle; ASD, atrial septal defect; RAA, right aortic arch; AAH, aortic arch hypoplasia; 45X, Turner Syndrome; T21, Trisomy 21; NND, neonatal death; QF-PCR, quantitative fluorescent polymerase chain reaction; array CGH, array comparative genomic hybridization; N, normal.

**Table 2:** Results of Pearson’s Chi-Square test.

Variable	Chi-Square Test Value	*p*-Level
DV	0.0	1.0
Umbilical drainage	4.114	0.127
PVS	7.199	0.027
Additional sonographic findings (fetal anomalies)	9.765	0.0075
Genetics	2.125	0.144

PVS: portal venous system.

## Data Availability

No new data were created or analyzed in this study. Data sharing is not applicable to this article.
